# Coevolution of Environmental Perception and Cooperative Behavior in Evacuation Crowd

**DOI:** 10.1038/s41598-018-33798-w

**Published:** 2018-11-05

**Authors:** Zehua Dong, Maoyin Chen, Yuan Cheng, Xiaoping Zheng

**Affiliations:** 1 Department of Automation, Tsinghua University, Beijing, 100084 China; 2 School of Automation and Electrical Engineering, Linyi University, Linyi, 276005 Shandong China

## Abstract

For the evacuation crowd of social agents, environment plays a big effect on the behavior and decision of the agents. When facing the uncertain environment, the behavior and decision of agents depend heavily on the perception of environment. Therefore, the cooperation between agents and their perception of environment may coexist during evacuation. Here we establish a mechanism to analyze the coevolution between the cooperation of agents and the perception of environment. In detail, we use a regular square lattice with periodic boundaries, where two payoff matrices are used to describe two kinds of games between neighbors in the safe and dangerous environments. For individual agent, its perception can be adjusted by interacting with neighboring agents. When the environment is generally considered dangerous, the fraction of cooperative agents keeps at a high level, even if the value of *b* is very large. When all the agents think that the environment is safe, the fraction of cooperation will decrease as the value of *b* increases.

## Introduction

It is known that the cooperation can be easily observed in nature and human society. For example, ants will share food and information with others, badgers and coyotes prey together, the human work together, and so on. It learns that cooperation may make a group play a great power, and get more reward than working alone. However, according to Darwin’s theory of natural selection, cooperation and individual selfish nature is contradictory. Therefore, how to understand the emergence and evolution of cooperative behavior remains a challenging task.

In order to understand how cooperative behavior evolves, the pioneering work is to establish the evolutionary game theory^1–5^, which provides a theoretical framework to address the conflict between cooperation and selfishness. In the past decades, the prisoner’s dilemma (PD) game^6^ was regarded as a paradigm for expressing the human relations in society and analyzing the cooperation. In a PD game, two players interact with each other each time. Both of them can choose to cooperate or defect. They get different rewards according to their choices. The reward for mutual cooperation is *R*, while the reward for mutual defection is *P*. If one player chooses cooperation and the other chooses defection, the cooperator receives the sucker’s payoff *S*, and the defector gets the defector temptation *T*. In the PD game, *T* > *R* > *P* > *S*. Hence one player can get maximum payoff by defecting. But if all the players choose defection, the total reward will be very small. On the contrary, cooperation can make the total reward increase, although cooperation is not an optimal choice for one player.

In addition, the spatial structure can also affect the evolution of cooperative behavior. In 1992, Nowak and May showed that structured populations via nearest neighbor interactions can promote cooperation, while the mixed populations lead to defection^7^. After that, some works placed PD into networks such as small-world^8,9^, scale-free world^10–12^, etc. Besides the spatial complex networks, many researches put forward different mechanisms that can promote the evolution of cooperation. For example, Nowak reviewed five rules (namley kin selection, direct reciprocity, indirect reciprocity, network reciprocity, and group selection) for the evolution of cooperation^13^. Additionally, evolutionary game with coevolutionary rules initiated by Zimmermann *et al*.^14,15^ offers a new way to promote the cooperation in a social dilemma. Coevolutionary rules may conform to realistic situations, where the strategies can evolve in time. However, some other factors such as links^16–19^, size^20,21^, mobility^22–24^, and age^25,26^, update at the same time, which can affect back the evolution of strategies.

Recently, coevolutionary multigames with different rules and structures have been discussed. Motivated by the fact that the same social dilemma can be perceived differently by different players, Wang *et al*.^27^ studied evolutionary multigames in structured populations. Chen and Wang^28^ adopted a stochastic learning updating rule to investigate the evolution of cooperation in the PD game on the small-world networks with different payoff aspiration levels. Wu *et al*.^29^ studied the evolution of cooperation in spatial PD games with and without extortion by adopting the aspiration-driven strategy updating rule. Traulsen *et al*.^30^ analyzed a cooperative game on the similarity between the players. Szolnoki and Perc^31^ introduced coevolutionary success-driven multigames in structured populations.

Except for the above mentioned factors, the environment is another factor strongly affecting the cooperative behavior. Note that human beings are actually in the environment all the time. Even for the same person, his/her behavior and decision may be differentially affected by different environments. In fact, the impact of the environment on people can not be ignored in the game. Some studies suggest that the environment will have a positive impact on the evolution of cooperation in the PD^32–35^. However, these studies only considered the objective environmental factors, such as links, mobility as mentioned above. What we want to know is whether people’s subjective perception of the environment will affect the evolution of cooperation. If the individual thinks that the current environment is dangerous, will he/she still be in a PD game?

In realistic situations, the ability of agents to perceive the environment is also different. This kind of heterogeneity may lead to the difference of the perception of environment. Even in the same scene, some agents consider that the current environment is relatively safe, but some consider it dangerous. This makes different agents take different strategies and show different behaviors in a game. In turn, the interaction between neighbors and their behavior can also affect their perceptions. This implies that there may exist some kind of coevolution between the environmental perception and the cooperative behavior. If it actually exists, but how to coevolve?

In this report, we consider this kind of coevlolution. We establish a mechanism of the coevolution of perception and cooperative behavior based on evolutionary game theory, where agents update their strategies and perceptions from more successful neighbors at the same time. From the evolutionary game theory and the coevolution mechanism, we study the cooperative behavior in a regular square lattice with periodic boundary conditions. First, we place the population in the lattice. Based on their current strategies and perceptions of the environment, agents choose payoff matrix to calculate their utilities. Then, each agent updates its strategy and perception from its random successful neighbors at a certain probability. Finally, using the Monte Carlo simulation, we record the proportion of cooperation and the proportion of agents who consider that the environment is safe. We find that when the environment is dangerous, the fraction of cooperation keeps at a high level.

## Results

In order to observe cooperation and perception in the population, we count the proportion of cooperation (*c*) and the proportion of safe agents (*s*) who consider that the environment is safe. From ‘Method’ section, the agents whose perception (*θ*) is less than the threshold (*θ*_*th*_) may think that the current environment is safe; otherwise, the current environment is considered to be dangerous.

**Figure 1:**
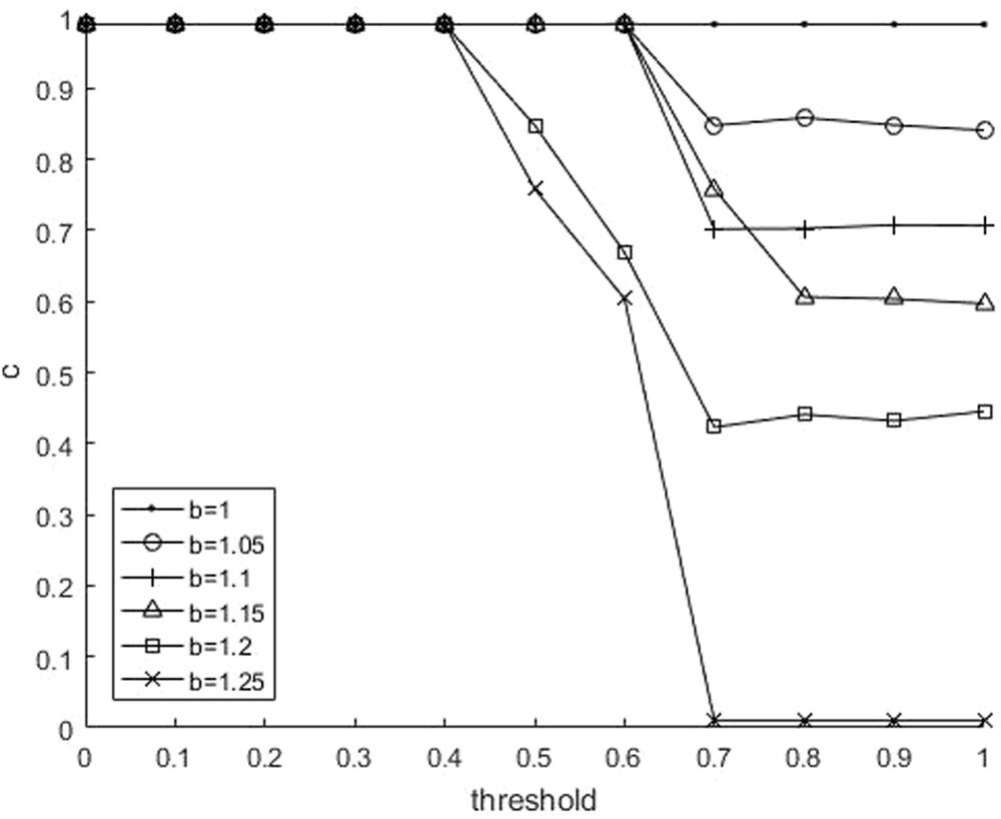
(Number of neighbors: 8). Relationship between the fraction of cooperation *c* and the threshold for parameter of *b*.

To begin with, we take a look at the effect of the threshold on the proportion of cooperation. Figure 1 shows the curve, where the cooperative proportion *c* changes with *θ*_*th*_ at different temptation parameter *b*. It is clear that for each curve, the cooperative proportion *c* is keeping at 1, and then gradually reduced to a stable value. The stable value decreases as the value of *b* increases, which is consistent with the traditional case^7^. Therefore, when the current environment is considered safe, people tend to do their own things, and do not care about others. This can be seemed as a typical PD game. On the contrary, when the current environment is considered dangerous, the person may think that his/her strength is limited, he/she can’t get much reward by himself/herself, and more people will choose cooperation to solve the difficulties together.

**Figure 2:**
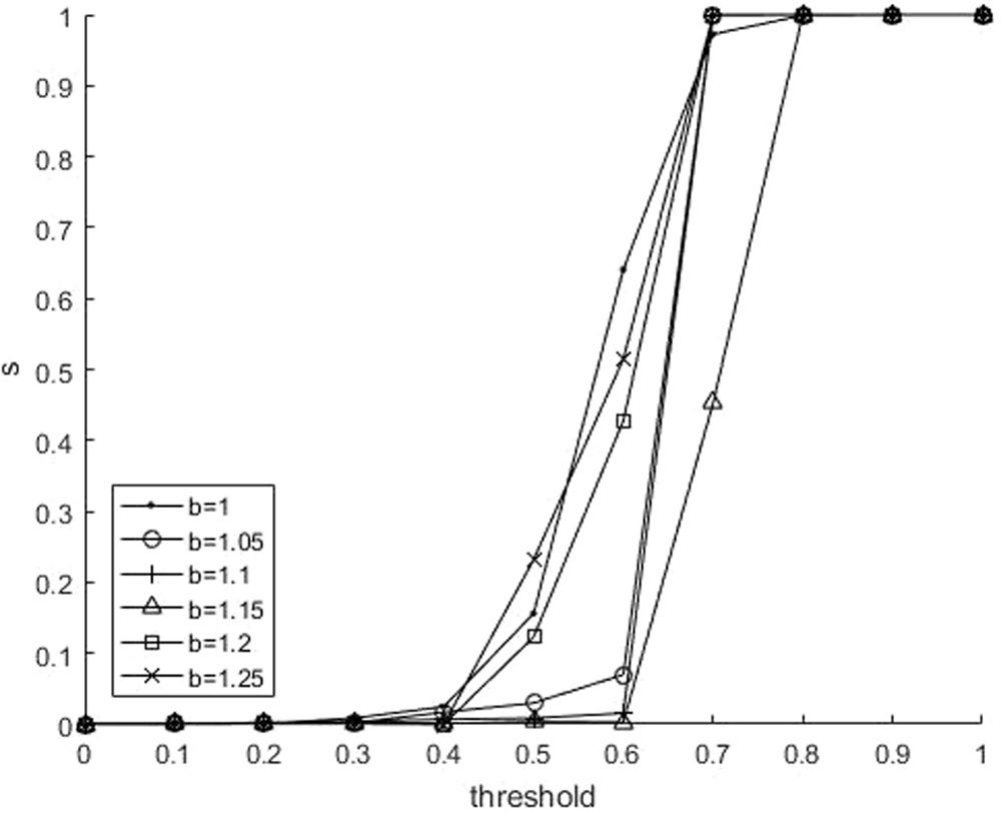
(Number of neighbors: 8). Relationship between the safe proportion *s* (namely the proportion of safe agents who consider that the environment is safe) and threshold for parameter of *b*.

Second, the relationship between *s* and *θ*_*th*_ is plotted in Figure 2. In the case of *b* = 1, two payoff matrices are very similar, and the game between agents is almost unaffected by their perceptions. When the threshold is small, all agents consider that the environment is dangerous. This may promote the cooperation between agents, which is consistent with Figure 1. When the difference between temptation *b* and 1 is small (for example, *b* = 1.05), compared with *b* = 1.1 and *b* = 1.15, the change of *s* is more similar to the case of *b* = 1, except that the sudden rise at *θ*_*th*_ = 0.63. This abrupt rise also appears when *b* = 1.1 and *b* = 1.15. In the case of *b* = 1, as previously mentioned, the game between agents is almost unaffected by their perceptions, so the whole system is insensitive to the threshold. However, when *b* > 1, the system is sensitive, and has a critical point. When *θ*_*th*_ = 0.63, there is a sudden increase of *s*. Figure 2 shows that that the less *b* is, the more *s* increases when the *s* is just increasing from 0 (*θ*_*th*_ = 0.30). When *b* = 1, the growth of *s* with respect to *θ*_*th*_ is almost linear, which implies that the whole system has little relationship to environmental perception. When *θ*_*th*_ is small, *s* is approximately equal to 0. In this case, almost all the agents agree that the environment is dangerous, which can assimilate the agents who consider the environment is safe. The closer to 1 the value of *b* is, the less difference between danger and safety has, *θ*_*th*_ has little effect on the system, and thus we observe the monotonicity. When *b* is large (for example, *b* = 1.2 and *b* = 1.25), the defection temptation makes more agents to choose to defect. Even if the threshold is not large (0.5–0.6), the agent who consider environment safe will be the majority. However, we observer that *s* ≈ 0 at *b* = 1.1 and *b* = 1.15. Therefore, the agents’ behavior indeed has an influence on their perceptions.

**Figure 3:**
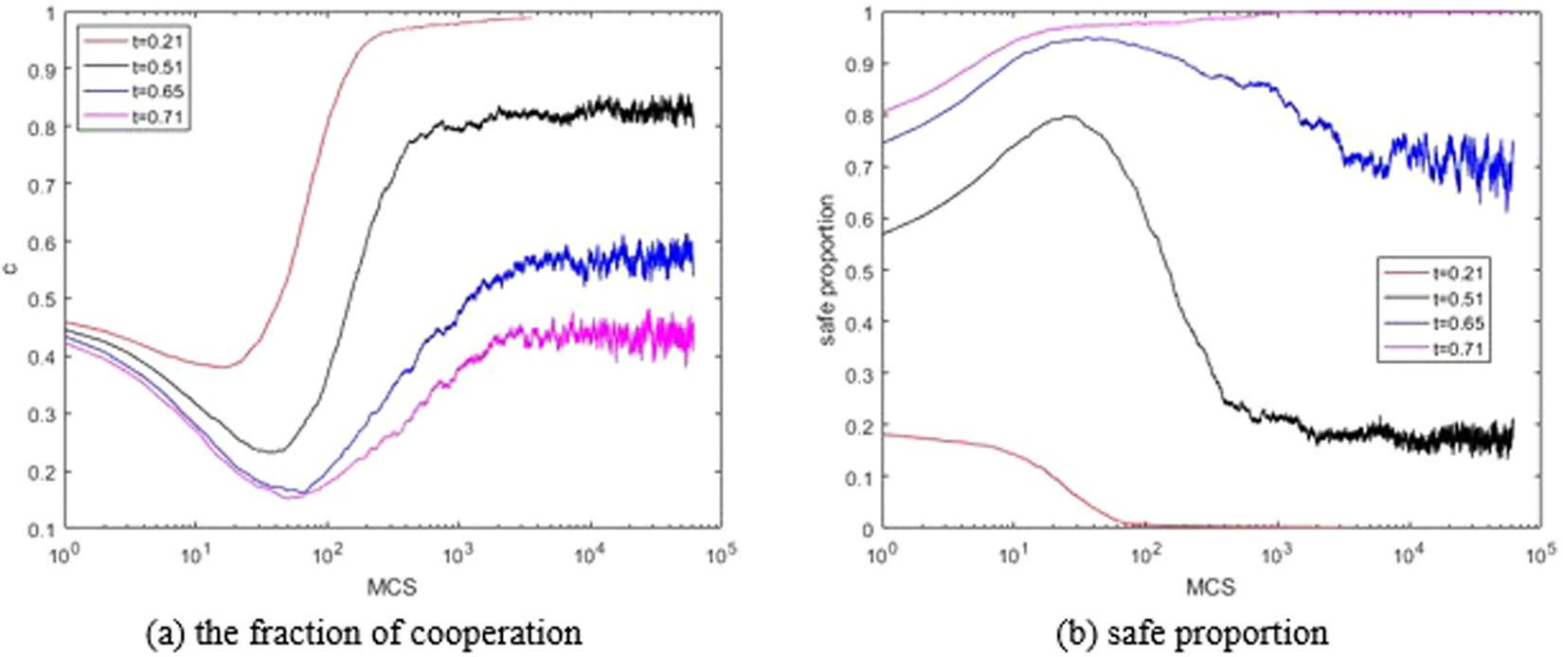
The fraction of cooperation *C* (left) and the safe proportion *s* (right) dynamics in 60000 steps for threshold (*b* = 1.2).

Figure 3 shows the dynamical changes for *c* and *s* in a run in the case of *b* = 1.2. It also shows that the larger the threshold *θ*_*th*_ is, the smaller cooperative proportion *c* is, and the larger *s* is. In this figure, we notice the negative feedback mechanism in the network^16^. The value of *c* decreases before 100 steps, and then increases. At the beginning, the numbers of cooperators and defectors are equal. In this PD game, the agent will choose to defect to get more payoff. After 100 steps, the proportion of defectors is so much that they can no longer earn more. At this time, both the cooperators and the defectors get the same reward when they play the game with a defector (*P* = *S* = 0). However, the reward for mutual cooperation *R* = 1, so the fraction of cooperation will gradually increase, and finally stabilize. When *θ*_*th*_ is moderate, we also observe the phenomenon of the decrease after the increase of *s* in Figure 2. For the agents who consider the environment dangerous, their best choice is to cooperate, while the others choose to defect. Hence, the greater the threshold *θ*_*th*_ is, the more the proportion of cooperation *c* decreases at the beginning. When *b* = 1.2 and *θ*_*th*_ ≥ 0.71, all agents consider that the environment is safe. This is actually the traditional case, that is, the proportion of cooperation *c* will be at a low level in the final.

**Figure 4:**
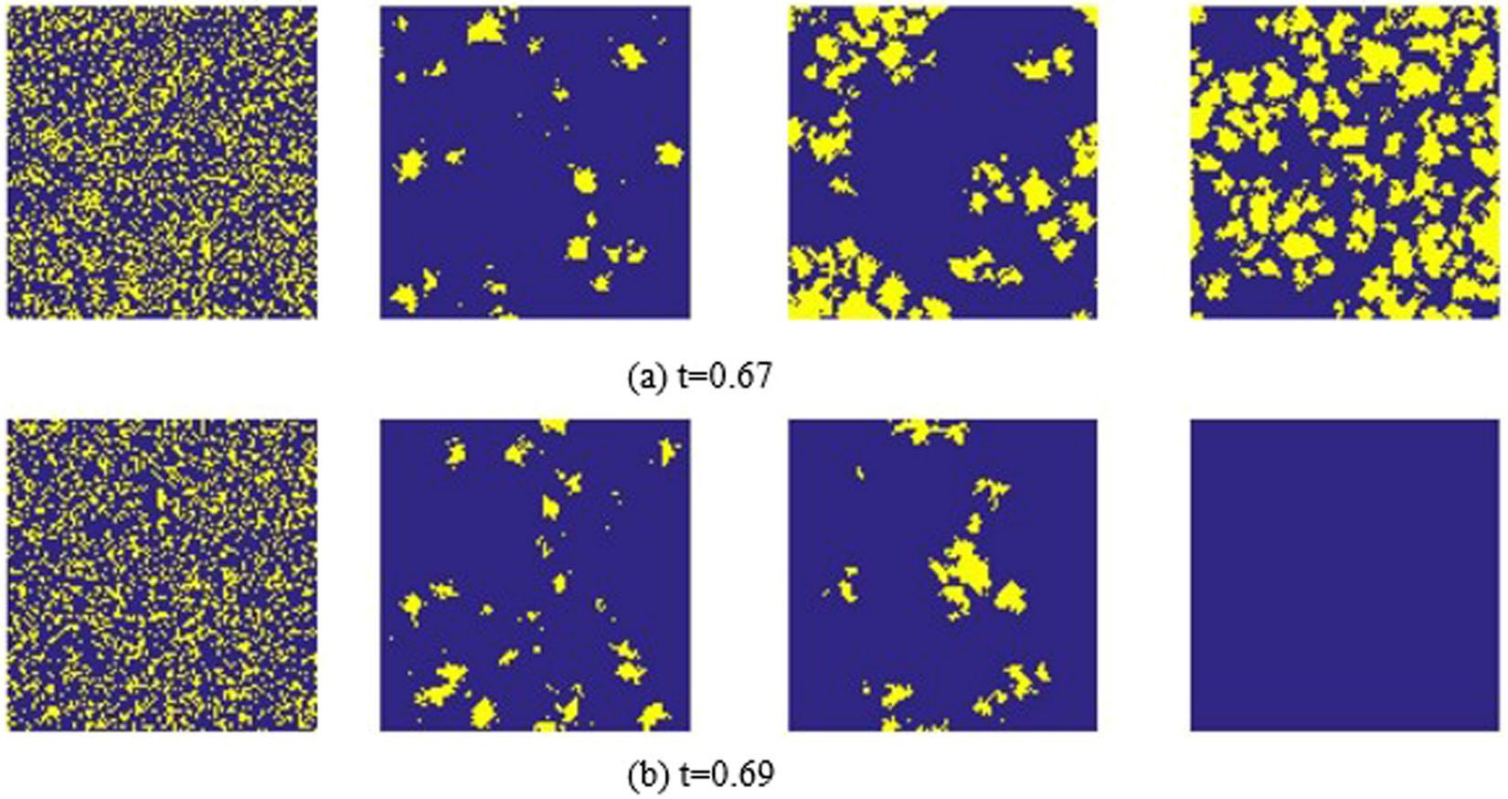
Distribution of *s* in step 0, 100, 1000, 60000 in 100 * 100 lattices (*b* = 1.2). From top to bottom, thresholds are 0.67 and 0.69, respectively. The yellow one represents the agent who considers the environment dangerous, the blue one represents agent who considers the environment safe.

Similar to Figure 3, Figure 4 also shows the negative feedback mechanism in the network. From left to right, Monte Carlo simulation steps are 0, 100, 1000 and 60000, respectively. From top to bottom, the thresholds are 0.67 and 0.69, respectively. Agents considering the environment safe (*s*) and agents considering the environment dangerous (*d*) are randomly distributed in the grid. At the 100th step, it is clear that *d* is reduced. At the 1000th step, the yellow area expanded again. Finally, at the 60000th step, the network is in a stable state. At *t* = 0.69, we notice that *d* = 0 in the population, implying all the agent consider that the current environment is safe. At *t* = 0.67, *s* and *d* coexist. As Figure 4 shows, the distributions of *s* and *d* are dispersed, that is, there is no obvious cluster of *s* and *d*. This increases the interaction area between *s* and *d*, and agents can switch between the two kinds of states, and the case in which *d* is surrounded and destroyed by *s* will not appear.

**Figure 5:**
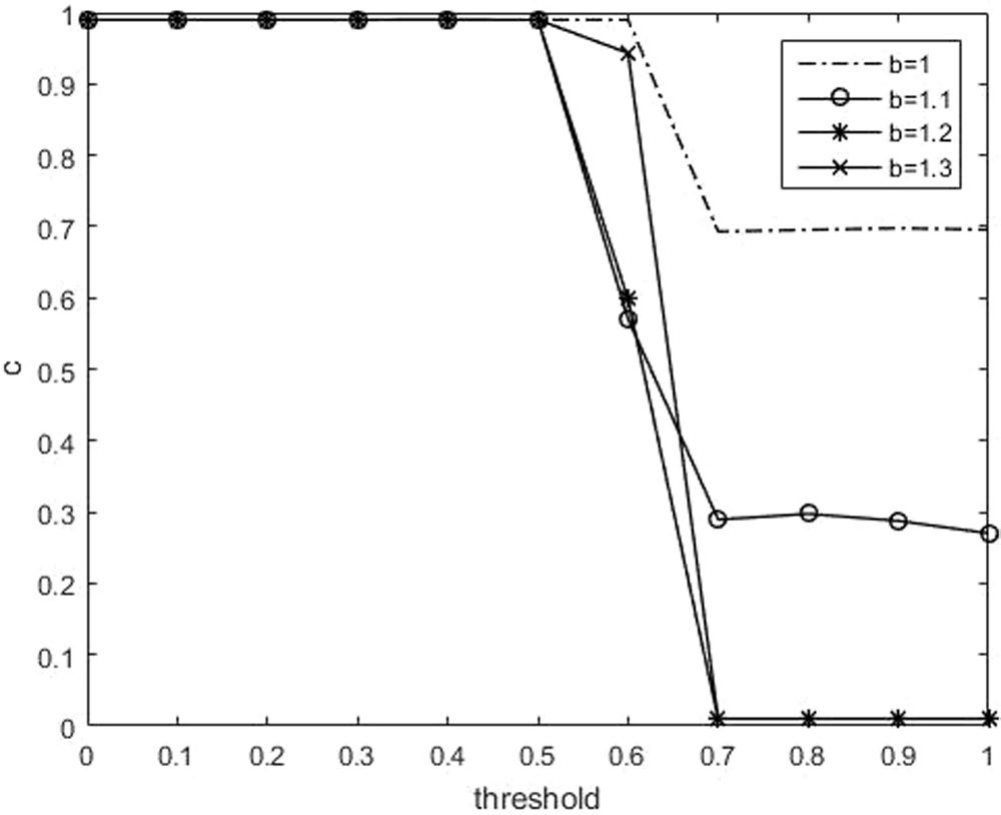
(Number of neighbors: 4). Relationship between the fraction of cooperation *c* and the threshold for parameter of *b*.

**Figure 6:**
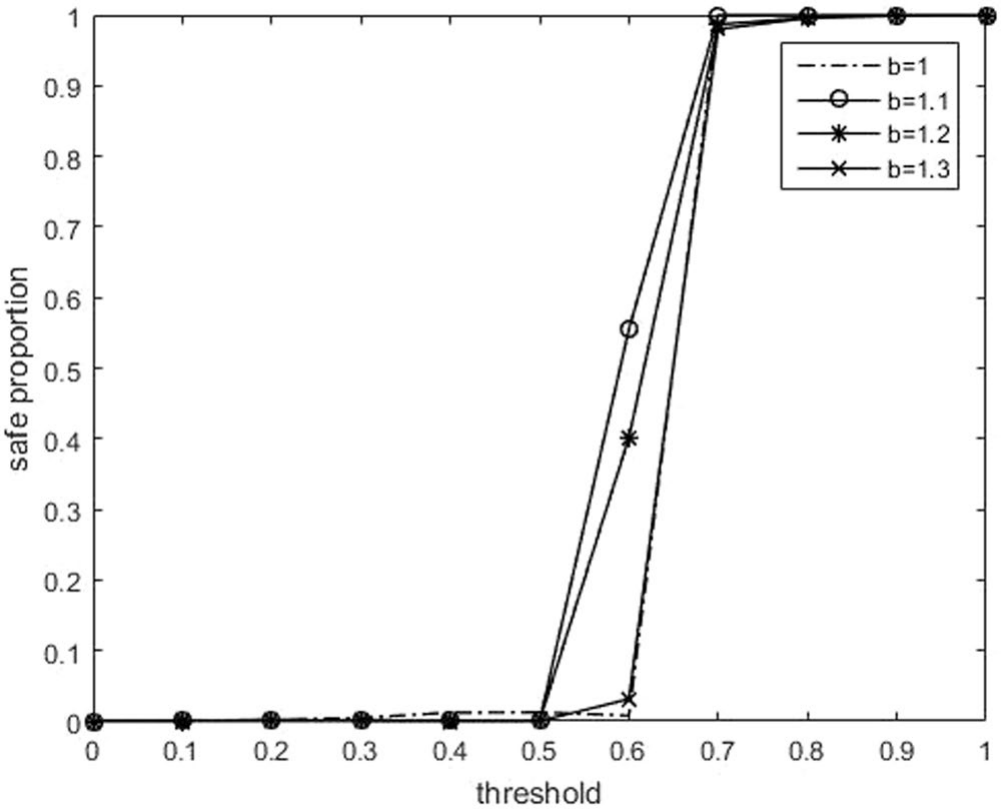
(Number of neighbors: 4). Relationship between the safe proportion *s* and the threshold for parameter of *b*.

To check the robustness of the results, we also did the experiments on the square lattice with degree = 4 and degree = 8. Figures 5 and 6 show the results when the number of neighbors is 4. They are consisitent with Figures 1 and 2, which shows the robustness of this mechanism.

## Discussion

In this report, we study the coevolution of environmental perception and cooperative behavior, specially their impact on the proportion of cooperation and agents who consider the environment safe. We find that the proportion of cooperation will increase greatly when the environment is dangerous. The agent’s perception of environment affects the strategy it will take, and in turn, the agent’s strategy and payoff also affect its perception. In fact, this kind of coevolutionary mechanism could be more realistic. In addition, we also discuss the negative feedback mechanism^16^ in this mechanism, explaining the evolution of the cooperative behavior. However, the ideal coevolution mechanism we build can’t reveal the essential relationship between perception and behavior. Moreover, all agents are assumed to follow the same rules in the simulation, which can’t reflect individuals’ diversity within the crowd.

We believe that studying the role of individual subjective factors in the evolution of cooperation is also necessary. In the literature, many works focus mainly on the objective factors such as link, age, mobility, network structure and so on. However, the impact of environment on its subjective perception is also a vital part that we can’t ignore during the crowd evacuation. In different circumstances, the individual’s subjective feelings are different, and then the decisions maybe not the same. In addition, even in the same environment, the subjective feelings of different individuals are not exactly identical. Being concerned about the subjective feelings of the individual can better understand the individual’s motivation to choose cooperation. When the individual makes a decision, the environment is one of the most important factors affecting the individual. Individuals may be persuaded by their neighbors in order to get greater earnings, or they may choose their own strategies based on the urgency of the current environment. Therefore, the study of the impact of environmental perception on cooperation may reveal the secret of the cooperation evolution.

We are going to improve our work in future. In this report, the cognition level on the safe environment is fixed in each run. That is, for all agents, the threshold for perceiving the safety of the environment is the same. However, if each individual has the same threshold, the heterogeneity owing to agents’ knowledge or experience can’t be reflected. So the threshold should be determined by a certain strategy, and should be random that follows the binary distribution, uniform distribution or gauss distribution. In general, the threshold can also be represented by an interval $[{\underline{\theta }}_{th},\,{\overline{\theta }}_{th}]$. If a neighbor’s perception is not in agent *x*’s threshold interval, then agent *x* will not choose to cooperate with this neighbor^36^. This is in line with the hypothesis of kinship selection, that is, when the individuals choose the cooperation object, they will give a preference to their similar individuals as much as possible to make their own genes can be passed. This is also consistent with the bounded confidence model in the opinion dynamics^37,38^.

Note that agents choose to cooperate with others because they can get the reward for mutual cooperation. However, some agents may show cooperative behavior without reciprocity^36^, and they help and donate to others even though they earn nothing, which can be called altruistic behavior. As far as we know, altruistic behavior in nature really exists. For example, male mantis will be eaten by the female mantis after mating. In human society, because of the educative background, people’s moral standards, the love and so on, people may also choose altruistic behavior. Since cooperation with reciprocity and altruism coexist in evacuation, we will consider in the follow-up work. In the game, not all individuals choose to cooperate or defect, and there are also some individuals can’t determine their decision. At the beginning of the game, not all individuals immediately choose reciprocity, altruism, or defection, and they may not know what to choose. This is a zero status, that is, the agent should wait and see what happened. The individual with zero status may join in the game at any time. In addition, it may be possible for them to withdraw from the game at any time because of the diminishing payoff. This assumption is more realistic than the assumption that everyone is involved in the game.

In ‘Method’ section, the update rule of the environmental perception uses the Fermi function, and the strategy’s update rule uses a linear rule. These two rules are generally accepted, in which Fermi function from the discrete selection model has some rationality. Note that update rules are very important for the model. However, except for the above two rules, are there other reasonable update rules that can optimize the system and make the model more reasonable? This question requires our future research.

Research on cooperation evolution is not only a fundamental problem, but also has a practical significance. For example, the crowd evacuation has always been an important part of social public security problem. In the evacuation process, the environment changes faster and more urgent, the environmental impact on people becomes more obvious. In addition, the behavior of the evacuated crowd is an important factor that affects the evacuation efficiency. Therefore, how to understand the evolution of the behavior is the key to solve the evacuation problem. Therefore, the research about environmental perception’s impact on cooperative behavior has a guiding significance to solve the evacuation problem.

To conclude, the agent’s perception of environment is an important attribute that affects the agent’s behavior, and may explain the evolution of the agent’s behavior in evacuation. We hope to further consider the environmental perception in the future.

## Methods

Assume that each agent can perceive the environment in a quantitative way. Let *θ*_*x*_(0 ≤ *θ*_*x*_ ≤ 1) be the quantitative perception value of environment by agent *x*. In order to study the effect of the environment, assume the agents’ cognition levels on the safe environment are identical. Here a constant cognition level on the safe environment is chosen to be *θ*_*th*_ > 0. For agent *x*, if its perception value of environment *θ*_*x*_ satisfies *θ*_*x*_ > *θ*_*th*_, the environment is considered safe; otherwise, the environment is dangerous. Note that the smaller *θ*_*th*_ is, the more dangerous the current environment is considered by agents. Therefore, some problems arise naturally: (1) Is it possible for each agent to change its perception value when interacting with other neighboring agents? (2) What is the effect of the changing of the perception values on the behavior of each agent?

Now we consider the above problems by introducing different kinds of games for safe and dangerous environment. For the safe environment, agents always do their own things and don’t need much help, so Nowak’s weak PD game^7^ is adopted. In this case, the defector temptation *T* = *b*_0_ > 1, the reward for mutual cooperation *R* = 1, the sucker’s payoff *S* and the punishment for mutual defection *P* equal to 0. For the case of dangerous environment, assume the agents who consider the current environment dangerous tend to look for help, so cooperation can increase the probability of escape. Hence, we set *T* = *b*_1_ < 1, *R* = 1, *S* = *P* = 0, which is a harmony game (HG).

In this report, we use a *L* × *L* regular square lattice with moore neighbor and periodic boundary conditions to place all agents. For the case of safe or dangerous environment, the perception of environment and the strategy in corresponding game will be updated by interacting with their neighbors. Specifically, an agent *x* selects its payoff matrix according to its perception of the environment, and calculates its total utility *U*_*x*_ by playing the game with all of its neighbors.Then, randomly selecting a neighbor *y* of *x*, if *U*_*y*_ > *U*_*x*_, *x* updates its perception by

1 $$\begin{array}{ll}{\theta }_{x}(k+\mathrm{1)}={\theta }_{x}(k)+\delta , & \,{\rm{if}}\,{\theta }_{x}(k) > {\theta }_{th}\,and\,{\theta }_{y}(k) > {\theta }_{th}\\ {\theta }_{x}(k+\mathrm{1)}={\theta }_{x}(k)-\delta , & \,{\rm{if}}\,{\theta }_{x}(k)\le {\theta }_{th}\,and\,{\theta }_{y}(k)\le {\theta }_{th}\\ p({\theta }_{y}(k)\to {\theta }_{x}(k+\mathrm{1))}=\frac{1}{1+{e}^{({{\rm{\Delta }}}_{x}-{{\rm{\Delta }}}_{y})/tt}}, & \,{\rm{otherwise}}\\ tt={\theta }_{th},\,{\rm{if}}\,{\theta }_{th}\ge 0.5 & \\ tt=1-{\theta }_{th},\,{\rm{if}}\,{\theta }_{th}\le 0.5 & \end{array}$$

where *δ* ∈ [0, 1] denotes the perception, and Δ_*x*_ = |*θ*_*x*_ − *θ*_*th*_|, Δ_*y*_ = |*θ*_*y*_ − *θ*_*th*_|. In the first and second equations of Eq. (1), if the perception of *x* and *y* are both greater than the threshold, *x* and *y* both consider the current environment safe. Then their perception values of *θ*_*x*_ will continue to increase by a small *δ*. If the perception values of *θ*_*x*_ and *θ*_*y*_ are both less than the threshold, *x* and *y* both consider the current environment dangerous. Then perception values of *θ*_*x*_ will continue to decrease by a small *δ*. Under this Tag-based mechanism^36^, a pair of agents who have the same cognition will strengthen their perceptions. Here we set *θ*_*x*_(*k* + 1) = 1 if *θ*_*x*_(*k* + 1) ≥ 1, and *θ*_*x*_(*k* + 1) = 0 if *θ*_*x*_(*k* + 1) ≤ 0. If there exist the opposite perceptions, where one thinks the environment safe and the other thinks it dangerous, *x* will copy the perception of *y* with a certain probability. Here the noise *k* in Fermi dynamics is “*tt*” in equation, which is the max distance between *θ* and *θ*_*th*_. When *x* thinks the environment safe and *y* considers it dangerous, we can calculate the distance(Δ_*x*_, Δ_*y*_) between the perception and threshold respectively, then discuss the difference between Δ_*x*_ and Δ_*y*_. Because the difference is no more than *tt*, the exponent is between 0 and 1. If an agent’s cognition is different with another’s, he/she may change his/her mind.

In the third equation of Eq. (1), we use the Fermi function^39^ to calculate the copy probability, and *k* denotes the noise. From this rule, the exponential term is determined by the difference between *δ*_*x*_ and *δ*_*y*_, where *δ*_*x*,*y*_ denotes the distance between the perception of agent *x*,*y* and the threshold *θ*_*th*_. That is to say, the probability of *x* learning *y* is mainly determined by this difference. If *δ*_*x*_ is greater than *δ*_*y*_, the probability is less than 0.5. Thus, *x* is not easy to be persuaded by *y*. If *δ*_*x*_ is smaller than *δ*_*y*_, the probability of *x* learning *y* is greater than 0.5; if *δ*_*x*_ = *δ*_*y*_, then the probability of *x* learning *y* is just 0.5.

Finally, agent *x* updates its strategy by comparing its utility with *y*. If *U*_*x*_ > *U*_*y*_, *x* will keep its own strategy. If *U*_*y*_ > *U*_*x*_, then *x* will copy the strategy of *y* with a certain probability^16^:

2 $$p({s}_{y}\to {s}_{x})=\frac{{U}_{y}-{U}_{x}}{8b}$$

where *s*_*x*_ denotes the strategy of the agent *x*, and the same as *s*_*y*_.

The simulations are realized within the framework of Monto Carlo simulation. Initially, agents are equally divided into cooperators or defectors. Environmental perceptions are assigned to agents at random, uniformly sampled from [0, 1]. An agent’s perception determines its payoff matrix, and it is affected by its more successful neighbors. In addition, we use the asynchronous update method. At each time step, randomly select an agent *x*, and update its strategy and perception in accordance with the above rules at the same time. The Monte Carlo simulation steps are 61,000. In the program, in order to simplify the parameters, we only reserve *θ*_*th*_ and *b*_0_, set *L* = 100, *b*_1_ = 0.9, and choose the learning rate *δ* = 0.001.

By changing the cognition level *θ*_*th*_, we observe the changes in the proportion of cooperation and safe agents who consider the environment safe. In order to reduce the disturbance, the program is replicated 10 times and the final result is averaged.
